# Time to unsafe sexual practice among cross-border female sex workers in Metemma Yohannes, North West Ethiopia

**DOI:** 10.1186/s12889-015-2035-4

**Published:** 2015-07-28

**Authors:** Lemma Derseh Gezie, Belaynew Wassie Taye, Tadesse Awoke Ayele

**Affiliations:** University of Gondar, P.o.box.196, Gondar, Ethiopia; Bahir Dar University, Bahir Dar, Ethiopia

**Keywords:** Female sex worker, Unsafe sex, Time to unsafe sex, Cross-border

## Abstract

**Background:**

Because of the nature of their work, female sex workers are at risk of sexually transmitted diseases. Cross-border areas are places where this situation becomes worse. In Ethiopia, there has been a serious scarcity of studies on the time at which unsafe sexual practice starts and on factors which determine the practice among female sex workers there. Therefore, this study aimed to fill this identified gap.

**Method:**

A total of 467 women who had been sex workers at least for three months prior to the resumption of the study were included. A structured and pre-tested questionnaire was used to collect data from July-August, 2010. Descriptive statistics was used to explore the data, and the Extended Cox-Regression model was employed to identify the predictors of time-to-unsafe sexual practice.

**Result:**

The study participants were followed for 6, 643 person-months. The overall incidence density of unsafe sexual practice was 44.71 persons per 1000 persons-months. The hazard of unsafe sexual practice increased by 3.0 % every month (p-value =0.040) due to problem-drinking. Those female sex workers with familiarized clients had a two-fold hazard of practicing unsafe sex compared to their counterparts (AHR = 1.94 95 % CI 1.49, 2.53). The predominant sexual client type and the work place of sex workers were the other significant predictors of unsafe sexual practice.

**Conclusions:**

The incidence of unsafe sexual practice was found to be high among sex workers in the cross-border area. Time-to-unsafe sexual practice was significantly associated with female sex workers’ status of familiarity with their clients, predominant sexual client type, their work place, and the interaction term of time and problem-drinking. Interventions need to be made on these controllable social and behavioral characteristics to help sex workers extend the duration of their safe sexual practice beyond the time they will quit sex work.

## Background

Since HIV/AIDS was first reported in 1981, nearly 30 million people have died of causes related to it, and approximately 34 million people are currently living with the virus worldwide. Of all HIV cases reported, the majority (97 %), are from low and middle-income countries; sub-Saharan Africa, the hardest hit region, is home to more than two-thirds (69 %) of the people living with HIV, while it has only about 12 % of the world’s population [1].

Ethiopia is one of the highly hit countries in sub-Saharan Africa. Once, there were about 1.2 million people living with the virus. The adult HIV prevalence was 1.5 %, (1.0 % male and 1.9 % female) [2]. The situation is worse in cross-border communities for different reasons [3].

Cross-border areas attract people from a variety of regions and countries with different cultural backgrounds and sexual experiences. Because of this situation, people living in cross-border areas are highly infected with STIs/HIV. Also, government and non-government organizations responsible for health care services might not have given sufficient attention to these border communities. This problem is likely to be more serious in African borders where health facilities and related infrastructure are not well developed. Moreover, some of the borders in the continent are experiencing civil unrest which can exacerbate public vulnerability to STIs/HIV. Furthermore, substances and drugs which are known to intensify the spread of STIs/HIV are highly abundant in these border areas [4–8].

In a manner similar to the other parts of the globe, the number of Female Sex Workers (FSWs) is growing in Ethiopia. For instance, FSWs were 2.1 % and 2.9 % of the total adult women in Addis Ababa and the other urban areas of the country, respectively [9, 10]. The proportion of sex workers in border areas could be even larger. This is because a large number of women who migrate to border areas in search of different opportunities finally join sex work. One of the reasons, for example, is that while traveling abroad illegally, some of them would usually be deported to these border towns. The unexpectedly low wage rate they encounter in the border areas when compared to the information they had earlier is the other common reason for joining sex work. In either of these cases, such women usually end up as sex workers in these border towns [11].

It was estimated that a considerable number of men (37.4 %) had sexual intercourse with women who were neither their spouses nor cohabiting. Of these sexual acts, nearly 1 % were with FSWs who sexually served men concurrently. Thus, FSWs were highly affected by HIV/AIDS when compared to women in the other segments of the population [1, 2].

Unsafe sexual practices are the most commonly incriminated means for the spread of STIs, including HIV/AIDS even though there are other different modes of transmission. Unsafe sexual practices mainly include unprotected vaginal/anal and oral intercourse [12] and multiple sexual partners [2]. Due to the nature of their work, FSWs have numerous sexual partners; hence, in this study, a particular emphasis was put on unsafe/unprotected sexual practice. Other studies also show that unprotected vaginal intercourse is the most common mode of transmission of HIV in Ethiopia [2]. According to a study carried out on FSWs in seven urban centers in Ethiopia, the prevalence of unprotected sex was 12 % [13]. Another study on FSWs in three small towns in north-west Ethiopia (Koladiba, Chuahit, and Dabat) indicated that only 12.8 % used condom consistently [14].

In Ethiopia, a number of studies [9–11, 13] were conducted on the risk of sexual practice on FSWs; however, none of them addressed the time-to-event of unsafe sexual practice. The majority of these studies were restricted only to the central districts where the context is totally different from border areas with regard to risky sexual practices [5]. Therefore, this study tried to determine the time-to-unsafe sexual practice and its predictors using different basic techniques of survival analysis on FSWs at Metemma Yohannes, northwest Ethiopia.

## Methods

A quantitative retrospective follow up study was employed to assess the time to unsafe sexual practice and its predictors among FSWs. The data were collected from July to August, 2010 at Mettema Yohannis, northwest Ethiopia, just at the point where the “Cairo-Cape Town Highway” enters the country.

According to the national census report, the town had 10,171 residents [15]. As there are a number of commercial farms around the town, tens of thousands of seasonal agricultural laborers live there temporarily. In addition, a report from the local administration indicated that a relatively large number (about 1200) of FSWs were living in the town. One reason for the disproportionate size was that the town is situated on the gate way abroad where female returnees usually joined sex work. The other reason was that women or sex workers from other areas migrated to the town as they were usually attracted by the market created by the seasonal massive agricultural laborers and foreigners who were their sexual clients [11].

In determining the sample size, we used a critical value associated with 95 % (i.e. 1.96), 3 % as a margin of error, and 12 % as a proportion of sex workers who used the condom consistently [13]. Adding 10 % to account for the non-response rate [16], we obtained the final sample size of 502. Taking the ratio of the population of FSWs (1,200) to the sample size (502), a sampling interval of 2 was used.

Based on the sample size determined, to trace and select FSWs, we started from the eastern side of the town and moved block by block assessing each and every house/establishment where FSWs were working. From the first sampling interval, the researchers randomly selected the first FSW as the first study participant. Then, the data collectors took every other FSW starting from that first sample participant until the required sample size was met. When data collectors got more than one FSW in an establishment (hotel/bar), they used the alphabet (or the letter) of their name to complete the sampling frame in that establishment.

Six well experienced female diploma graduate nurses and a female supervisor with a BSc. degree in nursing were deployed to collect the data. They were selected from areas other than the study site and were given a training which included field practice at Enkoye Mesk, Gondar town, for two days. During the data collection, onsite supervision was made by the supervisor and the investigators.

The survival data was collected by asking each FSW the date on which she started sex work in the town. That particular time was taken as the starting point of the retrospective follow up study. Then each FSW was asked the date on which she practiced unsafe sex for the first time which was the event of interest. Therefore, the length of time measured in months (from the start of sex work in the town to the event of unsafe sex) was taken to be the survival time for those who had experienced the event of interest. Participants who were in sex work and had unsafe sex before they started sex work in Metema Yohannes were taken as left censored. Similarly, FSWs who did not have unsafe sex until the date of interview were considered as right censored.

The predictors of time to the event of unsafe sexual practices considered were socio-demographic variables, frequency of alcohol drinking, problem drinking, use of other substances, knowledge about unsafe sex, attitude towards unsafe sex, predominant sexual client type, and familiarization. Data collected on these variables were exhaustively edited and verified both in the field and during data processing.

### Definitions

#### Problem drinking

If an FSW gave at least one “yes” response to any one of the following four CAGE questions, then that particular sex worker would be considered as having problem drinking: Have you ever thought you should Cut down on your drinking? Have you ever been Annoyed by other people’s criticism of your drinking? Have you ever felt Guilty about your drinking? Have you ever had an Early morning drinking to steady your nerve. The validity and reliability of CAGE questionnaire was shown to be acceptable by other authors [17].

#### Familiarization

If an FSW responded positively to at least one of the following **5 F** questions, then it was considered as there was familiarization. The questions were: Is there a client that: **F**rquently visits you?, you serve for **F**ree?, one or both of you **F**allen in love?, **F**urnishes your house with equipment?, has been your boy **F**riend? [18–20].

Data were entered using the statistical software called SPSS version 20, and both descriptive and analytical methods were employed to analyze them on the same software. The number and proportion of FSWs was descriptively determined by their socio-demographic, economic, and behavioral variables. The incidence densities of unsafe sexual practices were determined by calculating the number of FSWs practicing unsafe sex per 1000 FSWs per month. The Kaplan Mere curve was employed to describe the hazards of unsafe sexual practice by their familiarization status.

To determine the effect of each covariate on survival time using the Cox regression model, the proportional hazard assumption was checked by examining the linear interaction of each covariate with time. In this regard, the assumption of proportional hazard was met for all independent variables except problem drinking. However, problem drinking was found to be statistically significantly time dependent (p-value < 0.001). As a result, the Extended Cox Regression model was fitted. In this study, a p-value of less than or equal to 5 % was considered as statistically significant.

Ethical clearance, permission, and informed consent were obtained from the Institutional Review Board of the University of Gondar, the local administration, and the study participants, respectively. Before starting the interview, we informed each participant that they had the right to withdraw from the study at any time. Confidentiality of data was maintained through anonymous questionnaire; and participants were later counseled by health professionals to avoid risky sexual behavior.

## Result

Five hundred two sex workers were selected randomly. Of these FSWs, 474 (94.4 %) were followed, retrospectively to provide the information. The average age of the FSWs was 21.9 ± 4.1 SD; the youngest and oldest FSWs were 15 and 38 years old, respectively. Similarly, the average age of women at first sex was 15.8 ± 1.89 SD. Of the 474 FSWs involved in the study, 303 (63.9 %) had unsafe sex (Table 1)

**Table 1:** Socio-demographic, economic and behavioral characteristics of FSWs, Mettema Yohannis, 2010, Ethiopia

Characteristics	Number (percent)
Age	
15-19	148 (31.22)
20-29	297 (62.66)
30-39	29 (6.12)
Ethnicity	
Amhara	404 (85.23)
Tigrie	54 (11.39)
Others	16 (3.38)
Religion	
Orthodox	454 (95.78)
Muslim	18 (3.80)
Protestant & Catholic	2 (0.42)
Earlier marital status	
Never married	209 (44.09)
Divorced	254 (53.59)
Widowed/separated	11 (2.32)
Educational level	
Illiterate	193 (40.72)
Primary education	117 (24.68)
Secondary education and above	164 (34.60)
Former place of residence	
Urban	328 (69.2)
Rural	139 (29.3)
Foreign (Sudan)	7 (1.5)
Work place of the FSWs	
Working in one’s own home (hut)	32 (6.75)
Work in rental home (hut)	268 (56.54)
Work in Bar/Hotel	156 (32.91)
Mobile FSW	18 (3.80)
Problem drinking	
None	359 (75.74)
Present	115 (24.26)
Familiarized person	
None	262 (55.27)
Present	212 (44.73)
Total	474 (100)

.

### Survival time until unsafe sexual practice

When the 474 FSWs were assessed retrospectively, 7 were found to be left censored. That was because they had already been engaged in sex work and used to practice unsafe sex before they came to the study area. Again, among these left censored sex workers, 6 had unsafe sex after they started the business in the study area, while the remaining one had safe sex (right censored). Therefore, only 467 FSWs were considered in the survival analyses of whom 297 (63.6 %) had already practiced unsafe sex, while the remaining 170 FSWs didn’t do that until the date of interview (right censored).

The shortest survival time until the event of unsafe sex occurred in the first month of the sex work, and this was experienced by three FSWs; the longest survival time was in the 44^th^ month which was experienced by one women. On the other hand, among the 170 right censored FSWs, the shortest censoring time was in the 7^th^ month, and the longest was in the 62^nd^ month. Forty-nine FSWs experienced unsafe sex before the end of the fifth month in their sex work; 92 FSWs experienced unsafe sex during the interval of [5, 10) months. The probabilities of practicing unsafe sex before the end of the 10^th^, 20^th^, and 30^th^ months were 0.48, 0.71 and 0.83, respectively.

The overall median unsafe sexual practice free survival time was 15.0 months (95 % CI, 13.29-16.71). However, FSWs having familiarized client had a median unsafe sexual practice free survival time of only 11.0 months, while it was 23 months for sex workers without familiarized clients. The log-rank test of these two groups was also statistically significant (p-value < 0.001).

**Fig. 1:**
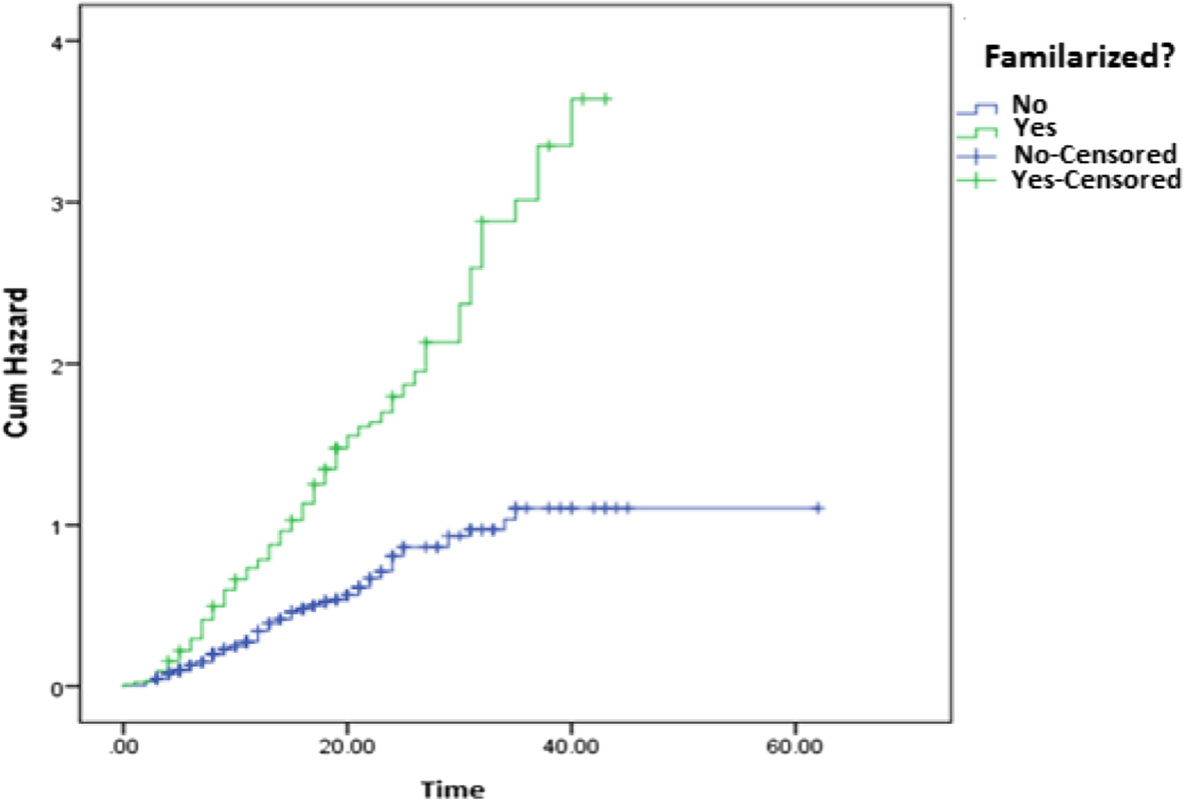
Cumulative hazard of unsafe sexual practice by familiarization status

The cumulative hazard function of FSWs who had familiarized sexual clients had a curve different from that of their counter parts. However, the two curves diverged constantly as time went on. Specifically, the cumulative hazard curve of each group increased with a different but constant rate for the earlier survival times (Fig. 1).

The median unsafe sexual practice free survival time was 13 months for FSWs with problem drinking and 17 months for FSWs without problem drinking. A log-rank test on these two groups also showed that there is a statistically significant difference between their median survival time estimates (p-value < 0.001).

### Multivariable survival analysis

A total of 9 candidate independent variables, including age, educational status, predominant sexual client type, problem drinking, level of alcohol use, familiarization, work place of FSWs, ethnicity, and monthly income were considered in fitting an Extended Cox Regression model from the survival data gathered. Before fitting this model, the proportional hazard assumption was checked for each independent variable using both subjective (informal) and statistical methods.

When each covariate was examined, only problem drinking was found to be statistically significantly time dependent. For instance, from Fig. 1, one could see that there were more or less constantly diverging cumulative hazard lines confirming the fact that the proportional hazard assumption was not violated for the covariate familiarization. Different statistical tests showed the same conclusion. For example, the uni-variate Cox regression model fitted using the interaction term of the covariate familiarization with time was not statistically significant (p-value = 0.943). However, the linear interaction term of time and problem drinking was taken as the 10^th^ independent variable to be considered in the multivariable extended Cox regression model because it was statistically significantly associated with time to event (p < 0.001).

The strategy employed to select the variables for the model was the Purposeful Selection Algorithm. Therefore, in the multivariable analysis, a significance level of 0.1 (as recommended by different authors) or a change of more than 20 % in the coefficients of others when a variable was omitted from the model were used in fitting the data [21, 22]. In this regard, variables including ethnicity, monthly income, and level of alcohol use in the multivariable analysis were neither statistically significant (p-value > 0.1) nor did their omissions considerably (>20 %) change the coefficients of others. As a result, these three variables were omitted from the final model. Therefore, including problem drinking and its interaction with time, a total of seven independent variables were included in the final model.

The overall person-time at risk of practicing unsafe sex (the sum of individual follow up times) was 6,643 persons-months, where ‘persons’ meant FSWs. The overall incidence density of practicing unsafe sex was 44.71 persons per 1000 FSWs-months. For FSWs aged 15–20, the incidence density of unsafe sexual practice was 47 FSWs out of 1000 FSWs-months. For mobile FSWs, the incidence of unsafe sexual practice was 96 FSWs per 1000 FSWs-months, while it was only 29 for FSWs working in bars or hotels (Table 2).

**Table 2:** Correlates of time until the event of unsafe sex among cross border FSWs, Mettema Yohannis, Ethiopia, 2010

Characteristics	Female sex workers’		Incidence	Adjusted HR	P- Value
	Number	Event	Density**	(95 % CI)	
Age					0.201
15-19 yrs	145	84	47	1.0	
20-29 yrs	293	198	46	0.843 (0.643, 1.11)	0.220
30-39 yrs	29	18	37	0.636 (0.373, 1.08)	0.095
Educational level					0.224
Illiterate	190	129	47	1.0	
Primary education	116	86	56	1.02 (0.77, 1.37)	0.872
Secondary and above	161	82	36	0.803 (0.605, 1.07)	0.129
Problem drinking					
None	355	194	37		
Present	112	103	73	1.06 (0.68, 1.68)	0.788
Familiarized persons					
None	259	106	28		
Present	208	191	169	1.94 (1.49, 2.53)	<0.001
Time*problem drinking	467	297	45	1.03 (1.02, 1.06)	0.040
Work place of FSWs					0.002
Work in one’s own home	31	18	36	1.0	
Work in rental house	264	206	53	1.21(0.74, 1.99)	0.453
Employed in bar/hotel	154	59	29	0.869 (0.51, 1.49)	0.607
Mobile sex worker	18	14	96	2. 91 (1.33, 5.52)	0.006
Predominant sexual client type					<0.001
Local Population	154	75	36	1.0	
Agricultural laborers	93	40	26	0.74 (0.0.49, 1.10)	0.131
Truckers/drivers	21	15	75	2.28 (1.28, 4.06)	0.005
Other Ethiopians	9	7	48	1.12 (0.50, 2.49)	0.783
Sudanese	190	160	61	2.14 (1.06, 3.88)	<0.018
Total	467	297			

**The incidence density of unsafe sexual practice per thousand FSWs per month

The hazard of unsafe sexual practice was found to be statistically significantly associated with variables, including familiarization, the interaction term of problem drinking with time, work place of FSWs, and predominant sexual client type. The hazard of practicing unsafe sex for FSWs having a familiarized client was 1.94 times that of FSWs without a familiarized client. At the beginning of the follow up time, (when time = 0), the adjusted effect of problem drinking was not found to be statistically significant. However, during the follow up time, the hazard of unsafe sex increased by 3.0 % every month.

The work place of FSWs was also an independent predictor of time to unsafe sexual practice. For instance, mobile sex workers had almost a threefold hazard of practicing unsafe sex compared to FSWs working in their own dwelling: (AHR = 2. 91 95 % CI 1.33, 5.52). Similarly, the predominant sexual client type was another predictor of time to event of practicing unsafe sex. Sex workers who had Sudanese and drivers as predominant sexual clients, had a twofold hazard of practicing unsafe sex compared to sex workers having local people as their predominant sexual clients: (AHR = 2.14 95 % CI 1.06, 3.88), and (AHR = 2.28 95 % CI 1.28, 4.06), respectively (Table 2).

## Discussion

In this study, 467 FSWs were followed retrospectively for a total of 6, 643 FSWs-months. The overall incidence density of unsafe sexual practice was 44.71 FSWs per 1000 FSWs-months. FSWs that did not have familiarized sexual clients had a median unsafe sexual practice free survival time of 23 months which was more than twice that of FSWs’ who had familiarized clients (11 months).

When adjusted for other variables, the hazard of practicing unsafe sex for FSWs who had familiarized sexual clients was almost twofold as compared to sex workers without any familiarized client, AHR 1.94 (95 % CI = 1.49, 2.53). Similarly, a study done in North Carolina indicated that if a male knew a FSW very well (familiarized), condom utilization rate would fall by 64 % [20]. A qualitative study conducted in Nigeria also indicated that it was not easy for FSWs to refuse unprotected sex to a familiarized sexual client [19].

These findings are of course justifiable because as FSWs get familiarized with their clients, they may not have the courage to refuse their familiarized clients who request unprotected sex. They may also be fatigued with the consistent use of condom for such familiarized clients who visit them frequently. As they stay longer with these sexual partners, FSWs may develop trust in their familiarized clients and could ignore the use of the condom. All these situations could affect the length of time that sex workers could stay practicing safe sex.

When adjusted for other variables, age, educational level, and problem drinking were not found to be statistically significantly associated with time to unsafe sexual practice. Even though we couldn’t get studies done on survival time until unsafe sexual practice, other studies made on unsafe sex reported a different result for educational level and problem drinking [11, 17]. Our findings in another study done on FSWs [11] highlighted that attending secondary and above educational level reduced the odds of practicing unsafe sex when compared to illiterate FSWs.

A study done in seven cities in Ethiopia [10] reported that attending secondary and above educational levels was independently associated with a reduced unsafe sexual practice. One possible explanation for these differences could be the fact that the current and other studies employed different outcome variables. In this study, the outcome variable is time-to-the event of unsafe sex, while other studies used the binary outcome which is unsafe sexual practice; education could have a different type of association with these two different outcome variables.

Problem drinking is not found to be an independent predictor of unprotected sex in the current study. However, according to our other study [11], it was independently positively associated with unsafe sexual practice. A study done in seven cities in Ethiopia [10] also showed that FSWs with problem drinking had a 50 % increased odds of unprotected sex compared with FSWs without problem drinking. Here, the justification of getting different results for problem drinking could be the same as to the reason we gave for education. However, its interaction with time was found to be an independent predictor of time to unsafe sex. More specifically, at the beginning of the follow up time, the adjusted effect of problem drinking was not statistically significantly associated with time to event. However, during the follow up time, the hazard of unsafe sex increased by 3.0 % every month (p-value =0.040).

This result could have different justifications. As mentioned above, problem drinking was found to be a time varying covariate, and FSWs’ status of problem drinking was taken cross-sectionally only once just at the end of the retrospective follow up time. However, a considerable number of FSWs might not have problem drinking at the start of the follow up time, while they could develop it later because of the nature of their work. Therefore, sex workers grouped by their last problem drinking status might not be significantly different with regard to survival time during earlier times, but could be different overtime. In line with this, the effect of problem drinking on survival time could be influenced by other factors, like dedication or commitment to practice safe sex which could be remarkably high when they start sex work but could diminish through time as they stay in their routine sex work. In turn, problem drinking could be one possible reason that could affect dedication and commitment to practice safe sex over time. Therefore, this could also be another possible reason why the effect of problem drinking was not statistically significant at the earlier survival times but significant afterwards.

The work place of FSWs was also found to be an independent predictor of time to unsafe sex (p-value ≤ 0.002). Mobile sex workers had almost a threefold hazard of practicing unsafe sex as compared to FSWs working in their own home. However, FSWs working in rental houses or employed in bars or hotels were not found to be different from those working in their own dwellings. This could be due to the fact that practicing sex work by moving from place to place could make FSWs more vulnerable to coercion or to any other situation which could lead them into unsafe sex which affects the length of survival time. On the contrary, FSWs working in bars and hotels could be protected by hotel or bar owners/managers from coercion, or even they (FSWs) could help each other as they lived together in those establishments. This could be one possible reason why FSWs working in establishments were not found to be significantly different from those working in their own houses.

### Limitation of the study

The primary limitation of this study is its potential recall bias. Of course, efforts were made to help FSWs remember the month in which they practiced the first unsafe sex during their sex work period. However, there could still be miss reporting, especially by FSWs who had unsafe sex long before the data collection period.

### Conclusions

The incidence of unsafe sexual practice among FSWs was high. Time to unsafe sexual practice was significantly associated with FSWs’ status of familiarity with their sexual client, predominant client type, work place of sex workers and the interaction term of time, and problem drinking. Interventions need to be made on these controllable social and behavioral characteristics to help FSWs extend their duration of safe sexual practice beyond the time they will quit sex work.

## Abbreviations

AIDS: Acquired immunodeficiency syndrome
STIs: Sexually transmitted infections
HIV: Human immunodeficiency virus
FSW: Female sex worker
AOR: Adjusted odds ratio
AHR: Adjusted hazard ratio
